# Carpenter’s Human Physiology

**Published:** 1853-01

**Authors:** 


### CARPENTER'S HUMAN PHYSIOLOGY.

The great popularity attained by Dr. Carpenter as a writer on Physiology renders any complimentary notice of his works quite a thing of supererogation. His standing is at least as high in our country as it is at home, and it is perfectly safe to say that no physiological writer in the English language has as many readers. Work after work has appeared from his prolific pen, and of the one before us, edition after edition, and each successive one has proved so excellent that the public has come to expect with interest whatever bears his name. He makes it a point to keep up with the progress of the science and to cause his works to reflect all the improvements in it; and withal the style in which he clothes his matter is so clear, graceful, and elegant, that one reads his volumes with delight.

His works are repositories of facts. He indulges but little in speculation, and devotes not much space to the conjectures of physiologists.

The present is the fifth American edition of Dr. Carpenter's Human Physiology, and has been remodelled to an extent which renders it almost a new work. He admits that in the progressive maturation of his views he has come to look upon many topics in a light very different from that under which he had previously regarded them—an admission which every writer on medicine must make who lives long and keeps his mind open to conviction. All that relates to our art and science is progressive, and he only can keep pace with them who is not too proud to confess that his judgment is fallible.

The additions which the author found it necessary to make to this work has swelled it into a volume of upwards of a thousand pages, and this may afford the reader some idea of the rapidity with which all the departments of our profession are advancing. The execution of their part by the publishers, Blanchard & Lea, is in the best style of American art. Any reader who desires a treatise on physiology may feel himself entirely safe in ordering this, as containing a full and faithful account of the science as it is.

